# Structure‐Photoprotective Capacity Relationship of Phenolic Hydroxyl, Methoxy, and Ethenyl Linker Moieties of Phenolic Acids

**DOI:** 10.1002/cbdv.202501604

**Published:** 2025-09-12

**Authors:** Sara Ghazi, Hoda Tavakoli, Enock Omakele, Luc J. Martin, Mohamed Touaibia

**Affiliations:** ^1^ Faculty of Science Université de Moncton Moncton New Brunswick Canada

**Keywords:** anti‐free radicals | hydroxycinnamic acids | solar protection factor | structure–activity relationship | ultraviolet radiation

## Abstract

As constituents of many plants, polyphenols can neutralize free radicals. By inactivating free radicals, skin homeostasis can be restored, which may prevent further damage and premature skin aging. To prevent the damage caused by UV exposure, photoprotection is becoming increasingly important. Plants known to be rich in polyphenols have been investigated for their use as sunscreens. The synergistic effect, which is due to the specific composition of plant extracts, is a disadvantage for applications such as large‐scale sunscreens. The great diversity of polyphenols present in such plants can be an obstacle to the identification of the most effective polyphenol families. Investigating polyphenol families by family could provide more information than plant extracts with various polyphenols and a multitude of other molecules. In this study, we focused on the phenolic acids subclass. Eleven phenolic acids have been investigated for their potential use as sunscreen. According to its Boots Star rating or to its critical wavelength, *p*‐coumaric acid shows good potential for use as a sunscreen especially for exposure to UVB radiation. The absence of cytotoxicity of coumaric acid on normal human dermal fibroblasts (NHDF), even at high concentrations, further validates the potential use of this acid in sunscreen formulations.

## Graphical abstract

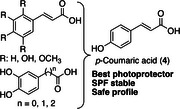

## 1 Introduction

Prophylaxis is an important element in the protection against skin damage caused by exposure to UV rays [1]. Sunscreens contain molecules or mixtures of molecules that can reflect, absorb, or even diffuse UV rays. The use of sunscreens with ingredients, such as zinc and titanium oxides, may result in negative health effects, including carcinogenic effect [2, 3, 4]. In addition to potential effects on human health, sunscreens also have an environmental impact, such as damage to coral [5]. Even the food chain can be disrupted by these sunscreens, because chemical UV filters, such as oxybenzone, have been found in various species of fish [6]. The use of natural solar screens can be a good alternative that is being explored more and more. Many plant extracts have been explored for their use as sunscreen [7]. Plants known to be rich in polyphenols have also been investigated for their use as sunscreen [8].

As constituents of many plants, polyphenols have the ability to neutralize free radicals. By inactivating free radicals, skin homeostasis can be restored, which may prevent further damage and premature aging of the skin [9]. As polyphenols can absorb UV in a broad spectrum, these molecules were reported for their protective effect against skin damage. Several extracts of whole plants or plant parts (seeds, leaves, stems, and roots) containing polyphenols have been investigated for their photoprotective effects [10]. Some extracts were enriched in flavonoids or polyphenols and were either natural UV filters or had synergistic photoprotective effects [10]. The synergistic effect, which is due to the specific composition of each extract, can be a disadvantage for applications such as large‐scale sunscreens. The great diversity of polyphenols present in such plants is a real obstacle to the identification of the most effective polyphenol families. Investigating polyphenol families by family could provide more information than plant extracts with various polyphenol families and a multitude of other molecule families.

**FIGURE 1:**
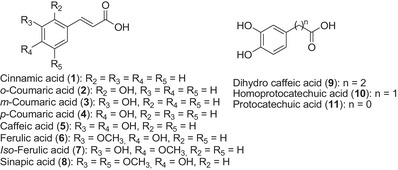
Cinnamic acid and its related analogs investigated as sunscreen.

In this study, we focused on the phenolic acid subclass. Phenolic acids are the main polyphenols produced by plants. These compounds have various functions and are extremely important in plant–microbe/symbiosis interactions [11]. From cinnamic acid (**1**) to protocatechuic acid (**11**) (Figure 1), 11 phenolic acids have been investigated for their potential use as sunscreen.

These acids and their derivatives are ubiquitous in the plant kingdom [12, 13, 14, 15]. Cinnamic acid (**1**) and its natural and/or synthetic analogs have been tested for various biological activities [16]. Among the 16 lignin model compounds, cinnamic (**1**), coumaric regioisomers (**3**, **4**), caffeic (**5**), ferulic (**6**), and sinapic (**8**) acids were investigated for their UV‐shielding performance by Li et al. [17]. However, the comparison of the 16 molecules in this study does not lead to clear conclusions regarding the influence of hydroxyl and methoxy moieties on the UV absorption, as some compounds, although present in lignin, have significantly different structures and functional groups (alcohols, aldehydes, and acids). Furthermore, the effect of the ethenyl linker is not investigated with the 16 selected molecules as dihydrocaffeic (**9**), homoprotocatechuic (**10**), and protocatechuic (**11**) acids were not tested in this study [17]. Few polyphenols, including resveratrol, coumaric (**4**), caffeic (**5**), ferulic (**6**) acids, and some synthetic analogs, have been investigated individually for their potential [18, 19, 20, 21].

To the best of our knowledge, these specific analogs have never been systematically explored together for their effects as sunscreens. Our study complements previous research that investigated certain phenolic acids individually or in smaller series, because it allowed us to investigate the three key moieties (hydroxy, methoxy, and the ethenyl linker) simultaneously within the same set of tests of the series of 11 selected phenolic acids.

The SPF of each phenolic acid of the selected 11 acids was measured at the two wavelengths (UVB: medium wave 290–320 nm; UVA: long wave 320–400 nm) mimicking the solar UV spectrum.

The influence of the number and position of hydroxyl groups on SPF was examined by comparing cinnamic acid (**1**) with the three coumaric acid regioisomers (**2, 3, 4**) and caffeic acid (**5**). The impact of the methoxy group, as found in ferulic (**6**), iso‐ferulic (**7**), and sinapic (**8**) acids, was also assessed. Additionally, the effect of modifications, as well as the presence of a linker between the aromatic ring and the acid group, was investigated by comparing caffeic acid (**5**) with its analogs dihydrocaffeic (**9**), homoprotocatechuic (**10**), and protocatechuic (**11**) acids. To better understand the structural effect on their SPF, the free radical scavenging activities, p*K*_a_, and lipophilicity of each phenolic acid in the series were also measured. The acids with the most interesting SPF were incorporated into a base cream to investigate the SPF of these creams. Critical wavelength of each phenolic acid incorporated in the base cream was also measured. The best acid of the whole series was finally investigated for its synergistic effect with the 4‐methylbenzylidene camphor, a frequently used filter and a highly effective oil‐soluble UVB absorber [22, 23].

## 2 Results and Discussion

### 2.1 The Photoprotective Capacity

#### 2.1.1 Phenolic Acid Solutions

**FIGURE 2:**
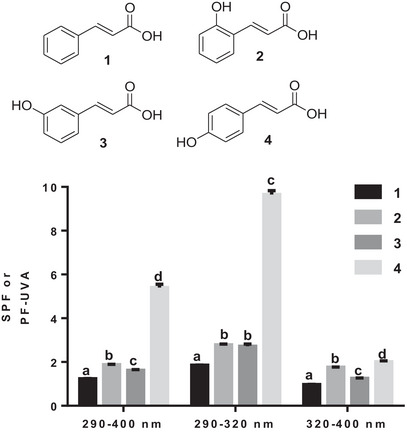
The photoprotective capacity of the first subseries of phenolic acids (**1**–**4**). Values are the means ± SD of three independent experiments, each performed in duplicate. For each wavelength range, bars without common superscript letter (a–d) are significantly different (*p* < 0.05) as determined by two‐way ANOVA of the non‐normalized data with subsequent Tukey's adjustment.

In the first subclass of acids, the presence of a single hydroxyl was investigated. As shown in Figure 1, the comparison between cinnamic acid (**1**) and *o*‐coumaric acid (**2**) reveals that the presence of a single hydroxyl moiety has only a slight effect on SPF in all three wavelength ranges (290–320, 290–400, and 320–400 nm). However, the position of the hydroxyl seems to be decisive for good SPF. As shown in Figure 1, *p*‐coumaric acid (**4**) is the best performing acid, particularly in the 290–400 and 290–320 nm wavelength ranges (Figure 2).

Directly and/or indirectly, exposure to UVB radiation can induce negative biological effects, including premature skin aging, oxidative stress, DNA damage, and multiple effects on the immune system [24, 25, 26, 27]. The electrostatic potential calculated due to the presence of the hydroxyl in the *para* position seems to be a determining factor, because compared with the other acids in the subseries, *p*‐coumaric acid had the lowest calculated electrostatic potential (0.0452 V) [28]. This potential was even lower than that of cinnamic acid (**1**) (0.0511 V). *p*‐Coumaric acid's (**4**) low potential combined with the strong electron‐donating effect of hydroxyl may explain its good performance as a sunscreen in both 290–400 and 290–320 nm wavelength ranges.

With the second subseries of acids, we investigated the effect on SPF of the number of hydroxyls and their position on the aromatic ring, as well as their replacement by a methoxy group. *p*‐Coumaric (**4**), caffeic (**5**), ferulic (**6**), iso‐ferulic (**7**), and sinapic (**8**) acids as well as their derivatives (esters, glycosides, and synthetic analogs) are examples of hydroxycinnamic acids with proven antioxidant properties [29]. Hydroxycinnamic acids's antioxidant activity stems from their ability to trap reactive oxygen species (ROS) due to the presence of free hydroxyl groups located at specific positions on the phenolic ring [30]. The nature and the length of the linker between the carboxylic acid and the aromatic ring of the phenolic acid moiety can play a crucial role in the antioxidant potency effect [31, 32].

**FIGURE 3:**
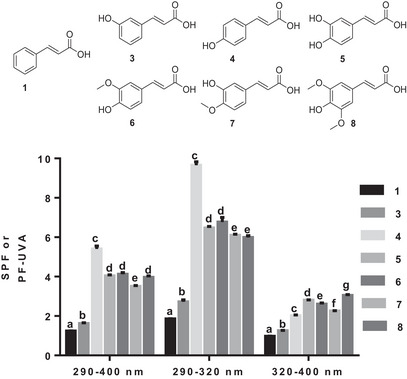
The photoprotective capacity of the second subseries of phenolic acids (**1** and **3**–**8**). Values are the means ± SD of three independent experiments, each performed in duplicate. For each wavelength range, bars without common superscript letter (a–g) are significantly different (*p* < 0.05) as determined by two‐way ANOVA of the non‐normalized data with subsequent Tukey's adjustment.

As shown in Figure 3, *p*‐coumaric acid (**4**) has a higher photoprotection property in the UVB range (290–400 and 290–320 nm) than the remaining acids (Figure 2). The presence of a second hydroxyl and its replacement by a methoxy group has no effect, because caffeic (**5**), ferulic (**6**), and iso‐ferulic (**7**) acids are all less efficient in the UVB range than *p*‐coumaric acid (**4**) (Figure 2). In the UVB range, caffeic (**5**), ferulic (**6**), and iso‐ferulic (**7**) acids were almost equivalent, with higher photoprotection property in the 290–320 nm wavelength range (Figure 3). The addition of methoxy moiety had no effect in the interval, because sinapic acid (**8**) was identical to ferulic acid (**6**) in the 290–400 nm wavelength range and even less effective in the 290–320 nm wavelength range (Figure 3). Although the SPF of the entire subset was less than 4, the SPFs of caffeic (**5**), ferulic (**6**), iso‐ferulic (**7**), and sinapic (**8**) acids were higher than those of the *m*‐coumaric (**3**), *p*‐coumaric (**4**), and cinnamic (**1**) acids (Figure 3). Even with the highest electrostatic potential after *m*‐coumaric acid (**3**) (0.143 V), ferulic acid (**6**) (0.0789 V) had a lower SPF than *p*‐coumaric acid (**4**), confirming that the presence of a methoxy group at position 3 has no effect on the molecule's SPF. Iso‐ferulic acid (**7**), which has an electrostatic potential (0.0462 V) very close to that of *p*‐coumaric acid (**4**) (0.0452 V), was less effective than the latter and ferulic acid (**6**), demonstrating the importance of the *para*‐hydroxyl position.

**FIGURE 4:**
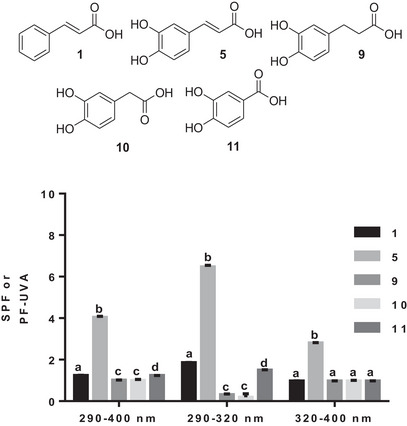
The photoprotective capacity of the third series of phenolic acids (**1**, **5**, and **9**–**11**). Values are the means ± SD of three independent experiments, each performed in duplicate. For each wavelength range, bars without common superscript letter (a–d) are significantly different (*p* < 0.05) as determined by two‐way ANOVA of the non‐normalized data with subsequent Tukey's adjustment.

With the third subseries, the effect of the linker between the aromatic motif and the carboxylic acid function on the SPF was investigated. The presence of two hydroxyl groups had an effect on the SPF, as shown by the comparison between cinnamic (**1**) and caffeic (**5**) acids (Figure 4).

The increased SPF observed with caffeic acid (**5**) can be attributed to the presence of both hydroxyl groups and the ethenyl (Figure 4). Hydrogenation of the ethenyl linker considerably reduced the SPF in all three wavelength ranges investigated, as shown by the SPF of hydrocaffeic acid (**9**). Reducing the number of linker carbons to one has no effect on SPF, because homoprotocatechuic acid (**10**) has the same SPF as hydrocaffeic acid (**9**) (Figure 4). Reducing the number of linker carbons to none, as for protocatechuic (**11**) acid, also drastically reduces the SPF compared to caffeic acid (**5**) (Figure 4). Protocatechuic acid (**11**) had a higher SPF than its analogs bearing a linker with two or one carbon property in the 320–400 nm wavelength range (Figure 4). The continuity of the aromatic system with the unsaturated linker seems to be crucial for better photoprotection, as shown by this third phenolic acid subseries. A reduction or shortening of the carbon chain of this linker reduces the in vivo photoprotective capacity of the phenolic acid.

#### 2.1.2 Phenolic Acids Incorporated in Base Cream

**FIGURE 5:**
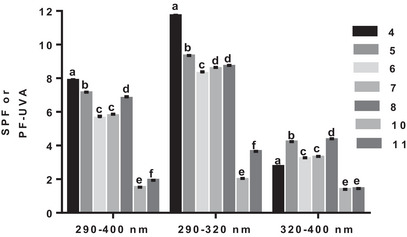
The photoprotective capacity of phenolic acids (**4–8**, **10**, and **11**) incorporated in a base cream. Values are the means ± SD of three independent experiments, each performed in duplicate. For each wavelength range, bars without common superscript letter are significantly different (*p* < 0.05) as determined by two‐way ANOVA of the non‐normalized data with subsequent Tukey's adjustment.

Phenolic acids (**4–8**, **10**, and **11**), with the highest SPF, were incorporated into a base cream for further investigation of their photoprotective capacity. As shown in Figure 4, the creams performed well in the 290–400 nm wavelength range and even better in the 290–320 nm wavelength range (Figure 5). In the first wavelength range, *p*‐coumaric (**4**), caffeic (**5**), ferulic (**6**), iso‐ferulic (**7**), and sinapic (**8**) acids had SPF between 6 and 8 and SPF between 8 and 12 in the second wavelength range (Figure 5). The cream with *p*‐coumaric acid (**4**) performed better in both 290–400 and 290–320 nm wavelength ranges (Figure 4). Creams with homoprotocatechuic (**10**) and protocatechuic (**11**) acids were the less effective in both 290–400 and 290–320 nm wavelength ranges (Figure 5).

In the third interval, creams with caffeic (**5**) and sinapic (**8**) acids were the most effective, with SPF around 4 (Figure 4). To determine the UV‐protected area with each cream with phenolic acids (**4–8**, **10**, and **11**), UVA/UVB ratios were calculated. Creams with caffeic acid (**5**), sinapic (**6**), and homoprotocatechuic acid (**10**) had the highest UVA/UVB ratio (Table 1), which indicates their photoprotective potential in the UVA range.

**TABLE 1:** UVA/UVB ratios, Boots Star rating, critical wavelength (*λ*c), and rating of creams with phenolic acids (**4–8**, **10**, and **11**).

Phenolic acids in base cream	UVA/UVB	Rating	*λ*c	Rating	pH
*p*‐Coumaric acid (**4**)	0.25	*	351	3	7.18 ± 0.02
Caffeic acid (**5**)	0.51	**	362	3	7.08 ± 0.02
Ferulic acid (**6**)	0.43	**	352	3	7.16 ± 0.03
Iso‐ferulic acid (**7**)	0.44	**	358	3	7.16 ± 0.01
Sinapic acid (**8**)	0.55	**	368	3	7.12 ± 0.04
Homoprotocatechuic acid (**10**)	0.53	**	392	4	7.13 ± 0.01
Protocatechuic acid (**11**)	0.41	*	389	4	7.12 ± 0.04

*Note*: Rating: *: ratio: 0.2 < 0.4; **: ratio: 0.4 < 0.6; 3: *λ*c 350–370 nm; 4: *λ*c ≥ 370 nm.

Having the UVA/UVB ratios, creams were classified between zero and five stars according to Boots Star rating criteria [33] (Table 1). All creams, except those with *p*‐coumaric (**4**) and protocatechuic (**11**) acids, were rated two stars, which means good protection in the UVA region. Using the criteria based on the critical lambda value of each cream recommended by the US Food and Drug Administration (FDA), creams with *p*‐coumaric (**4**), caffeic (**5**), ferulic (**6**), iso‐ferulic (**7**), or sinapic (**8**) acids were classified in category 3 as their critical wavelength (*λ*c) was in 350–370 nm range (Table 1).

Creams containing homoprotocatechuic acid (**10**) or protocatechuic (**11**) acids were classified as category 4, with the highest lambda near 400 nm. To avoid altering the skin's important role, the pH of creams is an important parameter to consider [34, 35]. As shown in Table 1, there was no significant difference between the pH of the creams obtained following the incorporation of each phenolic acid (**4–8**, **10**, and **11**), and all pH were around 7. Structural differences therefore have no effect on the pH of creams obtained with the different acids.

In view of the acidic character of the phenolic acids incorporated in the creams, we investigated the p*K*_a_ of these acids. With p*K*_a_ values around 4.6 and 4.4 of the same order as acetic acid, all the acids studied are rather weak acids, which does not prevent them from being used in cosmetics. As shown in Table 2, with a p*K*_a_ of 4.65, *p*‐coumaric acid (**4**) is the weakest of the series of acids incorporated into creams. Homoprotocatechuic acid (**10**) is the strongest acid in this series (Table 2).

**TABLE 2:** p*K*_a_, partition coefficient (Log *P*), and anti‐radical activity of phenolic acids (**4–8**, **10**, and **11**).

Phenolic acids	p*K*_a_ ^a^	log *P* ^b^	Anti‐radical activity (IC_50_)
*p*‐Coumaric acid (**4**)	4.65	1.26	29.15 mM (IC 95%: 24.19–32.13)
Caffeic acid (**5**)	4.58	0.93	10.14 µM (IC 95%: 8.80–11.69)
Ferulic acid (**6**)	4.58	1.36	36.61 µM (IC 95%: 30.85–43.33)
Iso‐ferulic acid (**7**)	4.53	1.39	2.17 mM (IC 95%: 1.89–2.48)
Sinapic acid (**8**)	4.53	1.31	16.71 µM (IC 95%: 14.36–19.44)
Homoprotocatechuic acid (**10**)	4.42	0.71	20.70 µM (IC 95%: 18.77–22.82)
Protocatechuic acid (**11**)	4.45	0.65	61.18 µM (IC 95%: 52.44–71.38)

^a^Calculated using Advanced Chemistry Development (ACD/Labs) Software V11.02 (1994 2023 ACD/Labs).

^b^Calculated with SwissADME [36].

The lipophilicity of molecules used in cosmetics can be a decisive factor in their optimal use, thereby contributing to good photoprotection in the case of sun creams [37]. For cosmetics, the lipophilicity of a material significantly affects the performance, stability, and absorption of the product. The partition coefficient (log *P*) provides valuable information about a compound's distribution between the lipophilic and hydrophilic phases. Lipophilic UV filters are used in sunscreens to ensure uniform dispersion and long‐lasting effectiveness [38]. As shown in Table 2, the lipophilicity of the selected acids ranges from 0.65 for the least lipophilic to 1.39 for the most lipophilic of the series (Table 2). Of all the acids of the series, homoprotocatechuic acid (**10**) is the least lipophilic, whereas iso‐ferulic acid (**7**) is the most lipophilic (Table 2).

In addition to their acidic character, phenolic acids have anti‐free radical properties due to their phenolic moiety. Combining the anti‐free radical effect with the photoprotective effect can be beneficial for use as a sunscreen. Being the only acid in the series with two hydroxyls, caffeic acid (**5**) had the highest anti‐antiradical capacity with the lowest IC_50_ (Table 2). All the other acids have interesting anti‐free radical activity that can be exploited at the same time as their photoprotective effect. Having only one hydroxyl and no other oxygen substituent, *p*‐coumaric acid (**4**) (29.15 mM) was the acid with the lowest antiradical capacity (Table 2). The addition of a methoxy at position 3, as with ferulic acid (**6**) (36.61 µM), clearly increases the molecule's antiradical capacity because the latter had greater antiradical capacity than *p*‐coumaric acid (**4**) (Table 2).

### 2.2 Synergistic Effect of 4‐Methylbenzylidene Camphor and *p*‐Coumaric Acid (**4**) on the Photoprotective Capacity

**FIGURE 6:**
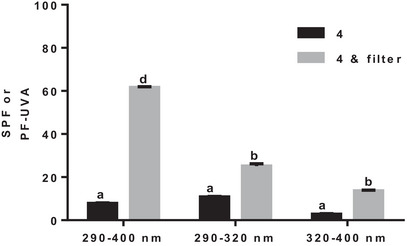
The photoprotective capacity of cream with *p*‐coumaric acid (**4**) alone or with 4‐methylbenzylidene camphor filter. For each wavelength range, bars without common superscript letter are significantly different (*p* < 0.05) as determined by two‐way ANOVA of the non‐normalized data with subsequent Tukey's adjustment.

Whether alone or when incorporated to a cream and according to its Boots Star rating or to its critical wavelength, *p*‐coumaric acid (**4**) is a phenolic acid with great potential for use as a sunscreen. To test its application as a sunscreen, its synergy when combined with 4‐methylbenzylidene camphor was investigated (Figure 6).

4‐Methylbenzylidene camphor (Neo Heliopan MBC) is a commonly used organic sunscreen active ingredient that is a highly effective oil‐soluble UVB absorber [22, 23]. As shown in Figure 6, the combination of the *p*‐coumaric acid (**4**) with 4‐methylbenzylidene camphor had a significant increase in all the wavelengths of the studied UV rays. The synergistic effect between the two molecules is especially important in the 290–400 nm wavelength range (Figure 6). As shown above, *p*‐coumaric acid (**4**) had an important effect in this same wavelength range. The cumulative π–π* interactions of the benzene rings of the two molecules can explain such increase in SPF [39]. The premise of the π–π* stacking interaction is that the two benzene rings are very close [40, 41, 42].

In the 290–320 nm wavelength range, the synergistic effect is not as strong as in the other two ranges. *p*‐Coumaric acid (**4**) can be used without this filter, which can have quite harmful biological and environmental effects [43]. *p*‐Coumaric acid (**4**) alone or incorporated in a cream was quite effective in this interval.

We also monitored the stability of *p*‐coumaric acid (**4**), both in its pure form and when incorporated into our base cream formulation. After 1 month of storage under ambient laboratory light exposure, we observed no significant change in SPF values, suggesting good preliminary stability under these conditions.

### 2.3 Cytotoxicity

**FIGURE 7:**
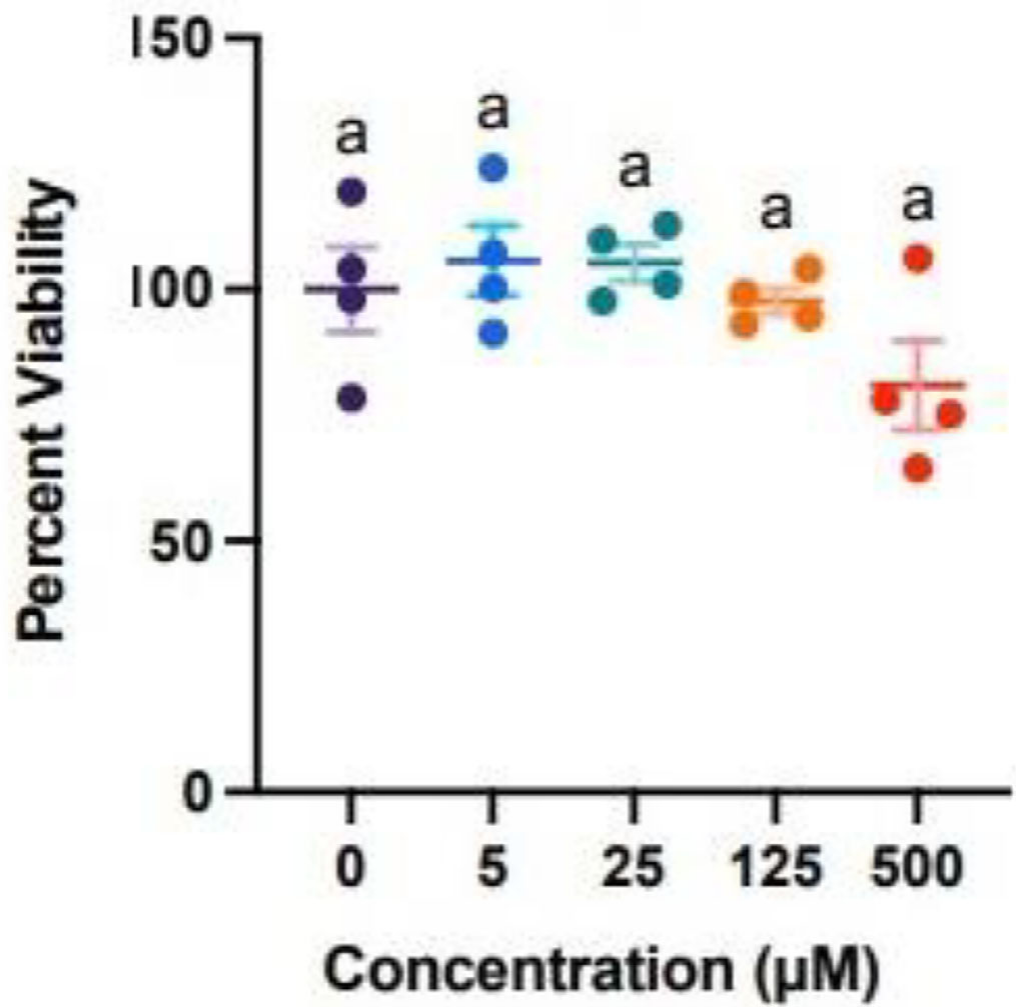
Adult normal human dermal fibroblast viability after treatments with *p*‐coumaric acid (4). Cells were incubated in the absence or presence of increasing concentrations (0, 4, 20, 100, 500 µM) of *p*‐coumaric acid (4) for 24 h, followed by determination of cell viability as described in Section 4. Experiments were repeated four times, and results are presented as percent cell viability over control (0 µM, DMSO only) (± SEM). Statistical comparisons were performed using a one‐way ANOVA followed by a Tukey multiple comparison test, where different letters indicate significant differences (*p* < 0.05).

As a major part of dermis, the fibroblasts are responsible for elasticity and hydration of the skin. Any substance that induces more than 30% of cytotoxicity may have harmful consequences for the skin [44]. *p*‐Coumaric acid (**4**) was remarkably safe for normal fibroblast cells, showing no cytotoxicity even at higher concentration (Figure 7). This lack of cytotoxicity of this acid could be attributed to its ability to avoid any interaction with specific biological targets, thus not disrupting the basic cellular functions. Given its anti‐free radical properties and photoprotective potential, as demonstrated above, *p*‐coumaric acid (**4**) can be a good candidate to be incorporated into a promising sunscreen.

When analyzed in structural alerts (https://solub.ochem.eu/alerts/home.do) [45], no alerts or specific features directly related to skin irritation were identified with *p*‐coumaric acid (**4**). However, as expected, the model did flag the α,β‐unsaturated carbonyl group, a common structural alert in toxicity prediction tools.

To further predict the risk profile of *p*‐coumaric acid (**4**) on skin, we used admetSAR 3.0 platform [46, 47]. Although *p*‐coumaric acid (**4**) is associated with a significant potential for skin irritation according to this model, its propensity for skin sensitization (38.8%) and phototoxicity (13.4%) is comparatively minimal (Table 3). Importantly, such predictions are made for the pure compound, whereas in a topical formulation, the molecule is never present alone but rather incorporated at controlled concentrations in a cream base, which markedly reduces the actual risk.

**TABLE 3:** In silico risk assessment of *p*‐coumaric acid (**4**).

Criteria	Percent risk assessment (%)^a^
Skin irritation	79.6
Skin sensitization	38.8
Phototoxicity	13.4

^a^Determined using admetSAR 3.0 [46, 47].

Therefore, both the experimental cytotoxicity results and the in silico predictions support the safety profile of *p*‐coumaric acid for topical application.

## 3 Conclusions

The use of plant extracts in cosmetics, particularly as sunscreen, is becoming increasingly widespread but can be limited by the complexity of the extracts themselves. The large number of molecules contained in an extract, combined with synergistic effects, prevents identification of the ingredient or even the mixture responsible for good photoprotection, adding to the impossibility of large‐scale production. Systematic investigation of molecules of a particular class can provide important information for the use of natural molecules as sunscreens. Whether alone or when incorporated to a cream and according to its Boots Star rating or to its critical wavelength, *p*‐coumaric acid (**4**) stands apart from its two regioisomers (*ortho* and *meta*‐coumaric acids) as well as all other acids for its potential for use as a sunscreen, especially for exposure to UVB radiation. The presence of a hydroxyl at position 4 of the phenyl ring as well as the ethynyl linker is crucial for effective UV protection, as demonstrated by our study. The absence of cytotoxicity of *p*‐coumaric acid (**4**) when incubated with normal human dermal fibroblasts (NHDF) further confirms its potential for use in sunscreen formulations given the risks of potential skin irritation or cytotoxicity issues. The identification of *p*‐coumaric acid (**4**) as an effective sunscreen could pave the way for the investigation of its synthetic derivatives, which may prove to be even more promising.

## 4 Materials and Methods

### 4.1 Materials

All phenolic acids and paraffin oil were purchased from Sigma‐Aldrich (Canada) and Eumulgin B2 (Ceteareth‐20), and cetiol HE (PEG‐7 glyceryl cocoate) were purchased from Trulux (Frenchs Forest, Australia). Stearic acid, triethanolamine (TEA), and xanthan gum (Keltrol BT) were purchased from Saffire Blue (Woodstock, ON, Canada). Glycerin was purchased from local pharmacy (Jean Coutu, Canada). Sodium propylparaben and sodium methylparaben were purchased from VWR (Canada). All purchased products had a purity of over 98% and were used without any purification or drying.

### 4.2 Photoprotection Assay

Absorption measurements, in the investigated wavelength intervals of ethanolic solution of phenolic acids (**1**–**11**) (50 µM), were obtained using a UV–Vis spectrophotometer (Genesys 10S UV–Vis, Quartz Cuvette: [volume: 3 mL, optical path: 10 mm]). SPF of each acid was calculated by the following formulas:

(1) $$\mathrm{SPF}\left(290-400\right)=\frac{\sum_{290}^{400}S\left(\lambda \right)\times \mathrm{EA}\left(\lambda \right)}{\sum_{290}^{400}S\left(\lambda \right)\times \mathrm{EA}\left(\lambda \right)\times T\left(\lambda \right)}$$

(2) $$\mathrm{SPF}\left(320-400\right)=\frac{\sum_{320}^{400}S\left(\lambda \right)\times \mathrm{EA}\left(\lambda \right)}{\sum_{320}^{400}S\left(\lambda \right)\times \mathrm{EA}\left(\lambda \right)\times T\left(\lambda \right)}$$

where EA (*λ*) is the erythemal action spectrum, *S* (*λ*) is the solar spectral irradiance, and *T* (*λ*) is the spectral transmittance value at the given wavelength. *S* and EA are constants [48].

(3) $$\mathrm{SPF}(290-320)=\mathrm{CF}\times \sum_{290}^{320}\mathrm{EE}(\lambda )\times I(\lambda )\times \mathrm{Abs}(\lambda )$$

where CF is the correction factor (=10); EE (*λ*) is the erythemal effect spectrum; I(λ) is the solar intensity spectrum; Abs (*λ*) is the absorbance at the given wavelength. EE (*λ*) and *I* are constants [49].

UVA/UVB ratio was calculated by the following formula [33]:

(4) $$\frac{\mathrm{UVA}}{\mathrm{UVB}}\mathrm{ratio}\;=\frac{\sum_{320}^{400}A\left(\lambda \right)\times \Delta \lambda /\sum_{320}^{400}\Delta \lambda }{\sum_{290}^{320}A\left(\lambda \right)\times \Delta \lambda /\sum_{290}^{320}\Delta \lambda }$$

where *A* is the absorption, and Δ*λ* is the measured wavelength interval.

The base cream into which each acid was incorporated was prepared as described [50], with a few modifications. To prepare 100 g of the base cream, all ingredients and their proportions [22] are listed in Table 4. To the cream base (25 mL), 1 mL of each phenolic acid (50 mM in ethanol) was added, followed by ultrasound‐assisted agitation for 1 min to obtain a homogeneous emulsion. Finally, the previous emulsion (1 mL) was diluted with isopropanol (25 mL). Absorption measurement, in the investigated wavelength intervals, of each isopropanol solution was obtained using a UV–Vis spectrophotometer as described above.

**TABLE 4:** Ingredients and proportions of base cream.

Ingredients	Percent by weight (g)
Paraffin oil	17
Cetiol HE (PEG‐7 glyceryl cocoate)	5
Butylhydroxytoluene	0.01
Stearic acid	5
Eumulgin B1 (Ceteareth‐12)	1.5
Eumulgin B2 (Ceteareth‐20)	1.5
Glycerine	4
Sodium propylparaben Sodium methylparaben	0.05 0.1
Keltrol BT (xanthan gum)	0.9
TEA	0.3
Distilled water	qsp 100

Abbreviation: TEA, triethanolamine.

Critical wavelength was calculated using MATLAB software. By choosing the best type of fitting curve, the integral amount and the area under the UV–Vis spectra of each cream were calculated. The critical wavelength (*λ*c) of each cream was estimated with the absorbance data (at 50 ppm) from SPF determination using the following formula [51]:

(5) $$\int_{290\;\mathrm{nm}}^{\lambda \mathrm{crit}}\mathrm{Abs}\;\left(\lambda \right)d\lambda =0.9\;\times \int_{290\;\mathrm{nm}}^{400\;\mathrm{nm}}\mathrm{Abs}\left(\lambda \right)d\lambda$$

where Abs (λ) is the mean absorbance at each wavelength, and dλ is the wavelength interval between measurements. The area under the absorbance curve (AUC) from 290 to 400 nm was 100%. The wavelength at which 90% of the AUC was reached was *λ*crit.

To evaluate the synergistic effect of 4‐methylbenzylidene camphor and *p*‐coumaric acid (**4**), 4‐methylbenzylidene camphor (1 g, 3.9 mmol) and *p*‐coumaric acid (**4**) (1 mL, 50 mM in ethanol) were added to the base cream (25 mL) to obtain the base cream with the filter. Finally, the previous emulsion (1 mL) was diluted with isopropanol (25 mL) before the absorption measurement as described above. The SPF of creams without and with 4‐methylbenzylidene camphor was calculated using Formulas (1–3).

### 4.3 pH Measurements

The pH of each cream containing phenolic acids was measured with a pH meter [52] (Orion model 290A).

### 4.4 Radical Scavenging Capacity

2,2‐Diphenyl‐1‐picrylhydrazyl (DPPH) (200 µL; 100 µM in methanol) was mixed with 200 µL of each acid at various concentrations (1, 5, 10, 20, 30, 50, 100, 150 µM) in methanol. The mixtures were kept in the dark for 30 min at room temperature. Absorbances at 517 nm of 100 µL of each mixture were then measured. The experiment was done in triplicate. The DPPH‐radical scavenging ability was calculated using the following formula [53]:

$$\begin{array}{ccc} & \%\;\mathrm{DPPH} & -\mathrm{radical}\;\mathrm{scavenging}\;\mathrm{ability}\;\\ & = & \left[\left(\mathrm{Acontrol}\;-\;\mathrm{Atest}\right)/\mathrm{Acontrol}\right]\;\times \;100 \end{array}$$

### 4.5 Cell Culture and Cell Viability

Adult NHDFs (NHDFa) were purchased from Sigma‐Aldrich and cultured in fibroblast growth medium 2 (C‐23020) (Millipore Sigma Canada Ltd., Oakville, ON, CAN) at 37°C and 5% CO_2_. At passages 6–8, cells were plated at 10 000 cells per well in a 96‐well plate, followed by incubation for 24 h. Treatments with increasing concentrations of *p*‐coumaric acid (**4**) (0, 5, 25, 125, 500 µM) were performed in Dulbecco's Modified Eagle Medium (DMEM) without serum and a consistent final concentration of DMSO of 1.67% (v/v). Following an incubation of 24 h, cell viability was determined using the Crystal Violet method as previously described [54].

### 4.6 In Silico Risk Assessment

The in silico risk assessment of *p*‐coumaric acid (4) was performed using structural alerts (https://solub.ochem.eu/alerts/home.do) and admetSAR 3.0 platform [46, 47], available at: https://lmmd.ecust.edu.cn/admetsar3/predict.php.

## Conflicts of Interest

The authors declare no conflicts of interest.

## Data Availability

The authors confirm that the data supporting the findings of this study are available within the article.
